# Introduction risk of fire ants through container cargo in ports: Data integration approach considering a logistic network

**DOI:** 10.1371/journal.pone.0313849

**Published:** 2025-02-07

**Authors:** Shota Homma, Daisuke Murakami, Shinya Hosokawa, Koji Kanefuji

**Affiliations:** 1 Big Data Technology Group, Port and Airport Research Institute, Yokosuka, Kanagawa, Japan; 2 Department of Statistical Science, Graduate University for Advanced Studies (SOKENDAI), Tachikawa, Tokyo, Japan; 3 Department of Fundamental Statistical Mathematics, The Institute of Statistical Mathematics, Tachikawa, Tokyo, Japan; 4 Marine Environmental Information Group, Port and Airport Research Institute, Yokosuka, Kanagawa, Japan; 5 Department of Interdisciplinary Statistical Mathematics, The Institute of Statistical Mathematics, Tachikawa, Tokyo, Japan; Jindal School of Environment and Sustainability, INDIA

## Abstract

Invasive alien species introduced to ports through cargo containers have destroyed the biodiversity worldwide. The introduction risk at ports must be estimated to control the early stages of invasion. However, limited data are available for this estimation in the introduction stage. Spatial statistical models have been used to address the lack of information by considering the observations of neighbors or integrating multiple data sources based on the assumption of spatial correlation. Unlike natural dispersal, methods to address these issues have not yet been established, because the spatial correlation between ports based on the geographical distance is not assumed for human-mediated species introduction through container cargo. Herein, we propose a multivariate conditional autoregressive model that considers a logistic network in order to integrate multiple data sources and estimate introduction risk. A relationship between locations based on logistics connectivity is assumed rather than the spatial correlation based on the geographical distance used in the past. Hierarchical Bayesian models integrating data through the network were implemented for two fire ant species (*Solenopsis invicta* and *Solenopsis geminata*) observed in Japanese ports. We observed that the proposed joint models improved the fit compared to conventional models estimated from a single dataset. This finding suggests that integrating data from multiple species or data types based on a network helps to address the lack of observations. This is one of the first studies to demonstrate the effectiveness of multivariate conditional autoregressive model in considering biological invasion networks and contributes to the development of reliable biosecurity strategies.

## 1. Introduction

Invasive alien species damage biodiversity and threaten human health, food security, and the global economy [1]. Ports and harbors are places where invasive species can be introduced through logistics. The risk of introduction at these points must be accurately assessed to regulate the early invasion stages. Human-mediated dispersal of alien species has been reported in all taxonomic groups worldwide, and contaminants in cargo containers are one of the main pathways for invertebrates [2, 3]. Eradicating these species is extremely difficult once they are established and their distribution is expanded. In particular, alien species are more likely to be established in highly artificial areas, where existing ecosystems have been destroyed [4]. Therefore, when considering invasion management at ports, such as monitoring and chemical eradication, the introduction stage is critical. Assessing the relative risk of introduction at each port is crucial for appropriately allocating resources for countermeasures [5].

Modeling logistic networks is necessary to estimate the introduction risk. The intensity of the introduction is considered a key factor affecting the invasion success in the propagule pressure hypothesis, and the introduction process is modeled by the volume of transportation [6]. Logistic networks also greatly affect invasion ecology [7–9]. Specifically, the introduction risk via container cargo may be affected by other locations through which the container transits, even when considering the volume transported from the regions where the target species is present [10]. Several stochastic models considering the networks have been proposed for modeling the introduction process of various taxa, including algae, fish, and insects [10–12].

Previous invasion models have helped understand the dispersion process with a large amount of data obtained after a species has been established. However, the observational data were limited to the introduction stage. Statistical models provide powerful tools for analyzing the relationship between introduction risk and numerous covariates from a small set of observed data [13]. This is because they allow the explicit consideration of observation errors and an accurate examination of the effects of environmental or ecological factors. In ecology, spatial statistic models have traditionally been used, and one of the main reasons is to better estimate events with low incidence rates using surrounding observation data [14]. These models consider the dependency between locations, without making them extremely complex. Owing to their ease of interpretation, conditional autoregressive models (CAR) are often used, particularly in ecology [14, 15].

As an expansion, integrating multiple ecological data sets has been reported to be effective using multivariate CAR (MCAR), which assumes a correlated spatial dependency. Developing modeling methods for combining available datasets is essential for a rapid response to invasion. Much effort has been focused on integrating designed surveys and presence-only data in recent years [16]. For example, integrating citizen science data and opportunistic surveys has been reported to improve prediction [17]. Although statistical models have accumulated knowledge on how to deal with rare events, the human-mediated introduction of alien species is not generally related to geographical distance, and methods have not been established to address the lack of information for modeling the introduction.

The model proposed in this study integrates data by considering logistics networks using MCAR. In recent years, the general concept of CAR as a network and its application to ecology have been discussed; however, it has rarely been used beyond the concept of space [14]. Owing to the network properties of the CAR, the model captures dependencies between ports based on logistics, not just distance or space. Moreover, the model combines data from connected ports and different data types, such as surveys and presence-only data, by assuming a correlated network structure. This study extends the application of this model to the human-mediated introduction of alien species, contributing to the effective management strategies against invasive species.

This study aimed to examine the MCAR model to assess the risk of the introduction of alien species. The model was tested on two fire ants, the red imported fire ant (*Solenopsis invicta*) and the tropical fire ant (*Solenopsis geminata*), which are native to South America and have recently been observed at Japanese ports in imported containers. The damage caused by the invasion of these species has been reported worldwide, and these species are in the introduction stage in Japan. Although the proposed model is applicable to general species, the main motivation for developing the model was to assess the introduction risk of these species. Using a hierarchical Bayesian model integrated with multiple datasets and MCAR, the effectiveness of the models was compared and verified based on the deviance information criterion (DIC) [18].

## 2. Materials and methods

### 2.1 Data

#### 2.1.1 Observational data

We analyzed the observational data for *S*. *invicta* and *S*. *geminata* aggregated and published by the Japanese Ministry of the Environment between 2017 and 2022. In Japan, *S*. *invicta* was first observed in a container imported into the port of Kobe in 2017 and has not yet been confirmed to be established [10]. Although *S*. *geminata* is known to be established on the southwestern islands of Japan (e.g., Ioto Island) at the time of the survey, it has yet to be confirmed to have colonized the mainland [19]. All data were identified by experts. Therefore, the observational records for these two species were interpreted as records of their introduction.

**Fig 1:**
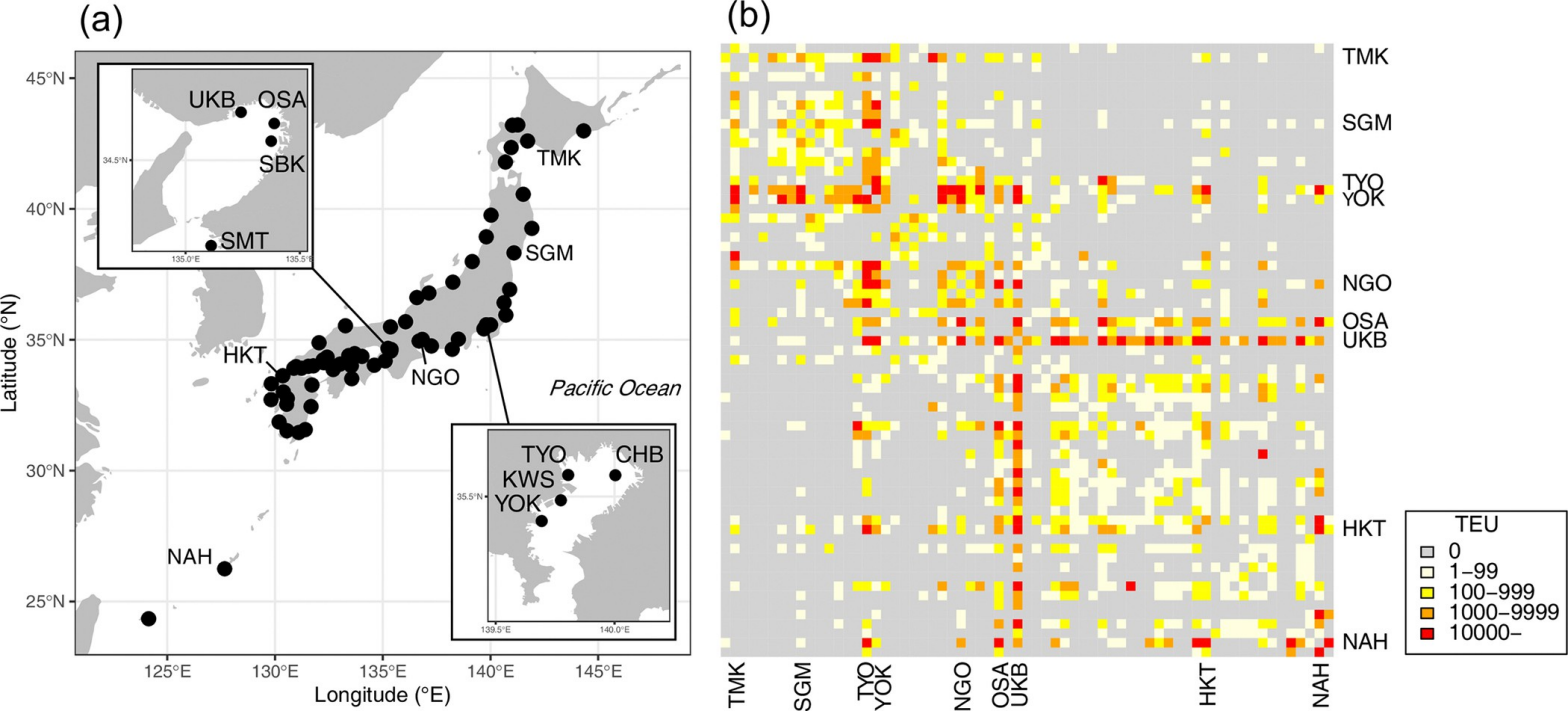
Location of the analyzed port and connectivity matrix. (a) Location of the port to be analyzed. The map data is obtained by the “mapdata” package v.2.3.1 in R [20], and the port location is based on the digital national information published by the Ministry of Land, Infrastructure, Transport, and Tourism of Japan. (b) Heatmap of connectivity matrix between domestic ports using container logistics. The order of the rows and columns corresponds to the numbers of the port in our analysis, and is generally sorted from northeast to southwest (S1 Table in S1 File).

We divided the dataset into two categories: presence/absence, obtained through a nationwide port survey, and presence-only. First, we tabulated the results of surveys conducted in 68 ports across Japan from 2017 to 2022, assuming that the introduction risk was stationary during this period (Fig 1A). The surveyed ports were selected based on them having regular container routes from the countries where these species were established (e.g., China and Taiwan). The port surveys were conducted primarily in summer and autumn. Visual surveys were conducted using bait traps and adhesive traps, depending on the situation. When suspicious individuals were detected, their identities were confirmed by experts, who reported whether they were *S*. *invicta* or *S*. *geminata*. We targeted 65 of the 68 ports that were surveyed at least six times (S1 Table and S1 Fig in S1 File), and the number of detections of *S*. *invicta* and *S*. *geminata* in each survey and the number of surveys were aggregated. The criterion was set such that if even a single individual of the species was observed, it was classified as “detected,” assuming that the detection probability was constant, regardless of the location. The results for each wharf were summarized as a single port. Consequently, 50 detections were obtained in 975 trials, 13–20 times for each port (S2 Table in S1 File). In this study, this dataset is described as the survey data.

The data published by the Ministry of the Environment include data discovered through independent surveys by port authorities and municipalities, and data discovered incidentally outside of port surveys. These supplementary data were considered less reliable because the extent of the effort put into these observations is unknown, making it difficult to pool them with the port survey. However, this type of data also includes observations outside the trap-based method; therefore, if a contaminated container is identified, it contains detailed information about the path taken by the container. This information may explain the variance in the survey data owing to the container network complexity. We extracted cases in which the port of entry was identified from these observation records and compiled them as introduction records at each port, distinguishing them from the survey data. In cases where a container was identified and found to have passed through multiple domestic ports, we counted the ports of transit and observation. In cases where species were observed in the follow-up surveys conducted after observation, we counted them as a single introduction event, according to the reported number in the original report list. A total of 115 observational records were obtained for both the species (S3 Table in S1 File). In this study, the dataset was described as presence-only.

#### 2.1.2 Explanatory data

We collected and organized data on the logistics volume of cargo containers and climate data as explanatory variables for the modeling. The countries where the target species were distributed were obtained from the Global Invasive Species Database [21, 22], and the annual mean number of containers imported from these countries to the target ports was obtained using port statistics from 2017 to 2022, collected and published by the Japanese Ministry of Land, Infrastructure, Transport, and Tourism (MLIT). To examine the impact of climate on the introduction risk, we obtained the minimum annual temperature from the World Climate [23] with a spatial resolution of 30 s. The minimum temperature for each port was assigned a value at the mesh to which it belonged, or in the adjacent land area. Port statistics were also used to construct a network of domestic container flows. We summarize the annual mean number of containers with a unit of twenty-foot equivalent unit exchanged between the analyzed ports based on statistics from 2018 to 2022.

### 2.2 Statistical model

#### 2.2.1 Observations

We use a hierarchical Bayesian model to estimate the relative risk of introduction at each port from the observed data. Let *Y*_*ijk*_ be the number of times that the species *k*∈{1,2} has been detected at port *i*∈{1,…,*N* = 65} in the port survey data (presence/absence data) *j* = 1 and the presence-only data *j* = 2.

The port survey data is assumed to obey the following binomial distribution:

$$Y_{i1k}∼\mathrm{Binomial}(n_{i,\mathrm{survey}},p_{ik}),$$

where *n*_*i*,survey_ is the number of surveys conducted at the port *i*. The parameter *p*_*ik*_ represents the detection probability, which indicates the potential risk of introduction of each species at each port, specified as

$$\mathrm{logit}(p_{ik})=\alpha_{1k}+\beta_{1k,1}{\mathrm{NC}}_{ik}+\beta_{1k,2}{\mathrm{MT}}_{i}+\theta_{i1k},$$

where *α*_1*k*_ is an intercept, *β*_1*k*,*l*_ is the *l*-th regression coefficient, and *θ*_*i*1*k*_ represents the spatial random effect depending on the network (see Section 2.2.2). Relative value of *p_*ik*_* represents the relative risk of introduction at each port. As covariates, we consider the number of containers imported to port *i* from countries where the target species *k* is present (NC_*ik*_) and the annual minimum temperature at port *i* (MT_*i*_).NC_*ik*_ is log-transformed, assuming that the introduction risk is moderated as the logistics volume increases. This nonlinear assumption was also used in models of species introduction using ballast water [13, 24]. Previous researches using ecological niche modeling predicted that the target species are less likely to be established in the northern part of Japan [25, 26], and that the annual minimum temperature is considered a significant factor in regard to the establishment of these species. However, the effect on the introduction risk throughout the year is unknown, and the minimum annual temperature was used to analyze these relationships.

For the presence-only data (*j* = 2), we assume a Poisson distribution with mean *λ*_*ik*_:

$$Y_{i2k}∼\mathrm{Poisson}(\lambda_{ik}),$$

$$\log(\lambda_{ik})=\alpha_{2k}+\beta_{2k,1}{\mathrm{NC}}_{ik}+\beta_{2k,2}{\mathrm{MT}}_{i}+\theta_{i2k},$$

where *α*_2*k*_,*β*_2*k*,*l*_,*θ*_*i*2*k*_ are defined similarly as *α*_1*k*_,*β*_1*k*,*l*_,*θ*_*i*1*k*_, and *λ*_*ik*_ represents the expected number of detections. This Poisson regression model is commonly applied to presence-only data for a relative risk model of alien species in ecology and disease risk mapping in epidemiology [17, 27].

We applied a stepwise model selection procedure to identify variables that significantly explained the data. We set the model described above as the full model and compared it with the two reduced models, each removing one variable. If the DIC of a reduced model increased by more than 2 compared with that of the full model, the variable was considered to contribute to the model. Otherwise, the reduced variable was excluded from the model. We also checked the result using Watanabe-Akaike information criterion (WAIC). Therefore, the MT variable was removed from the model (S5 Table in S1 File).

#### 2.2.2 Random variables

*Univariate model*. First, we modeled multiple data sources *j* and species *k* separately. For considering spatial dependency through the logistic network, we assumed the random variable *θ*_*ijk*_ to follow a conditional autoregressive (CAR) model [16]. We assessed spatial autocorrelation by interpreting the posterior distribution of the parameters of the random variables in the model. Although the CAR model has been widely used in ecology and other fields, it has rarely been extended to incorporate dependency through networks, such as logistic networks in ecology [15].

We use the Leroux model, which is a modified CAR model that can capture weak spatial dependency [28], expressed as follows:

$$\theta_{i}|\theta_{∼i}∼N\left(\frac{\lambda \sum_{i^{\prime }}w_{ii^{\prime }}\theta_{i}}{\lambda \sum_{i^{\prime }}w_{ii^{\prime }}+1-\lambda },\frac{\tau^{-1}}{\lambda \sum_{i^{\prime }}w_{ii^{\prime }}+1-\lambda }\right),$$

where ***θ***_~*i*_ = {*θ*_1_,⋯,*θ*_*i*−1_,⋯,*θ*_*N*_}, *w*_*ii*′_ is given by *α*×[the number of containers exchanged bidirectionally between ports *i* and *i*′] where *α* is a scaling factor detailed below, and *λ*∈[0,1] is the spatial dependency parameter, and *τ* is the precision parameter. Note that $N(\mu ,\;\sigma^{2})$ represents a normal distribution with mean *μ* and variance *σ*^2^. The distribution of ***θ*** = [θ_1_,⋯,θ_*N*_]^*T*^ yields:

$$\theta ∼N(0,\tau^{-1}{\left[\left(1-\lambda )I+\lambda \{diag(W1)-W\right\}\right]}^{-1}),$$

where **W** is a symmetric weight matrix (*N*×*N*) whose (*i*,*i*′)-th element equals *w*_*ii*′_ with zero diagonals, **I** is an *N*×*N* identity matrix, **1** is an *N*×1 vector of ones, and diag(**W1**) is a diagonal matrix with the row sum elements of **W**.

Matrix **W** is usually applied in binary form, where the (*i*,*i*′)-th element equals one if regions *i* and *i*′ are adjacent and zero otherwise. The scale of **W** affects the model fit [29]. Our weight matrix, which is based on container logistics, has a wider range of values than the binary matrix. Thus, the weight matrix was scaled to have a maximum value of 1 using *α*. In this transformation, the positive definiteness of the inverse covariance matrix of the multivariate Gaussian distribution, called the precision matrix, is preserved, and the joint distribution is proper. The resulting random variables ***θ*** have a map pattern induced by the dependence through a logistic network.

*Multivariate model*. This study considers the following integration models:

- Survey data *j* = 1 and presence-only data *j* = 2 for *S*. *invicta k* = 1,
- Survey data *j* = 1 and presence-only data *j* = 2 for *S*. *geminata k* = 2,
- Survey data *j* = 1 for *S*. *invicta k* = 1 and *S*. *geminata k* = 2.

The integration method described below is common across all the models. For simplicity, index *k* is omitted from the formulation in this section.

Random effects correlated across the datasets were introduced as a method of data integration. Such a correlation-based integration method has been reported to be more robust for the integration of low-quality data than other integration methods that assume shared parameters [30]. For data integration, we applied the MCAR prior [31–33]. For (i) and (ii), we define the MCAR prior as:

$$\Theta ∼N(0,\Lambda^{-1}⊗{\left[\left(1-\lambda )I+\lambda \{diag(W1)-W\right\}\right)}^{-1}).$$ (1)

Herein, ***Θ*** = [***θ***_1_^*T*^,⋯,***θ***_2_^*T*^]^*T*^, where ***θ***_***j***_ is a vector of random effects for *j*-th dataset and

$$\Lambda^{-1}=\left[\begin{array}{cc} \tau_{1}^{-1} & \rho /\sqrt{\tau_{1}\tau_{2}}\\ \rho /\sqrt{\tau_{1}\tau_{2}} & \tau_{2}^{-1} \end{array}\right],$$

where ⊗ is the Kronecker’s product, *τ*_*j*_ is the precision parameter, and *ρ* is the correlation coefficient between the datasets. The information on the random effect of each dataset is transferred through ***Θ***. Integration model (iii) is implemented in a similar manner in (1).

### 2.3 Model implementation and evaluation

We estimated 11 models including the three multivariate models. Each of the 11 models is a model with the explanatory variables selected using the model selection process described in Section 2.2.1. As base models, four models without random effects (GLM) were estimated for the two species and two data types. Four univariate CAR models, including the random effects of the network with Leroux CAR, were estimated for two species and two datasets. Two multivariate models integrating survey and presence-only data (MCAR-dt) were estimated for each species, and a model integrating survey data across species (MCAR-sp) was estimated.

All models were fitted using an approximate Bayesian inference method, which is an Integrated Nested Laplace Approximation (INLA) proposed by Rue, Martino, and Chopin [34] that is faster but as accurate as the Markov chain Monte Carlo method [35]. This approximation was performed in R v4.4.1 [36], using packages INLA v.24.5.10 [34]. Our model was not implemented in INLA. Therefore, we defined models applicable to INLA by using “rgeneric” function. Some multivariate CAR models that differ slightly from the Leroux model have already been implemented using the INLAMSM package, and we implemented the models by modifying them [32]. The models were compared using the DIC [18]. In addition, we supplemented the WAIC to improve its validity.

*Prior of hyperparameters*. We assumed non-informative prior distributions for the hyperparameters. For all the linear coefficients of the fixed effect, a N(0,1/0.001) was assumed. The gamma prior with a shape of 1 and scale of 100 was applied to *τ* of univariate models, and uniform prior with a range of 0 to 1 is assumed to *λ* for all Leroux CAR models. For the **Λ** of multivariate models, Wishart distribution was assumed with the degree of freedom of 4 and scale matrix of 2×2 identity matrix, following the specification for the precision matrix of the multivariate normal distribution in INLA. This distribution is the conjugate distribution of the precision matrix of the multivariate normal distribution and the multivariate gamma distribution form. Assuming the identity matrix to be a scale matrix, the distribution is relatively non-informative [32, 33].

## 3. Results

### 3.1 Observational data analysis

First, the observational data was examined. The port surveys were conducted an average of 14.8 times, with a minimum of 13 and a maximum of 20 times during the analysis period, at the 65 selected ports (S2 Table in S1 File). According to the port survey, *S*. *invicta* was observed at least once in 11 of the 65 ports. The port of Tokyo (TYO) had the highest number of detections (8 of 19). The average rate of detection per survey at each port was 2.02%, and the maximum was 42.1% for TYO. Similarly, *S*. *geminata* was found once or more in 11 of the 65 ports surveyed. The highest detection number was six at the port of Yokohama (YOK). The average detection probability per survey at each port was 2.1%, and the maximum was 33.3% for YOK.

In the aggregated presence-only data, *S*. *invicta* was observed at least once at 11 ports. The maximum number of observations during the analysis period was 13 for TYO. Moreover, *S*. *geminata* was observed at least once at 13 ports, and the maximum number was 19 at TYO. TYO and YOK are among the ports with the highest volumes of imported containers in Japan, suggesting a relationship between the introduction and imported container volume.

**Fig 2:**
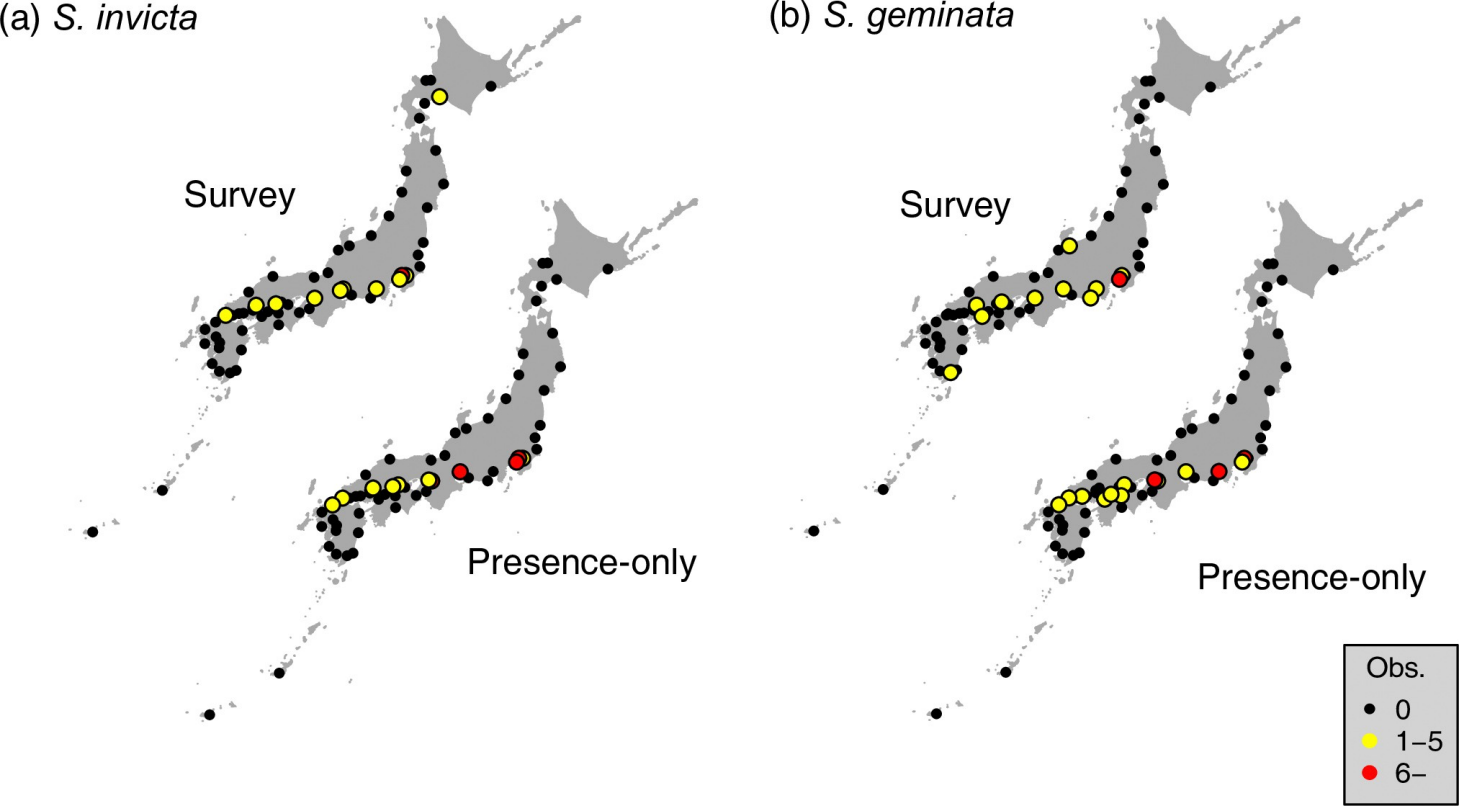
Observations map. Number of observations of *S*. *invicta* (a) and *S*. *geminata* (b). The different data types, including surveys and presence-only data, are displayed separately in each panel.

Next, we visualized the observational data (Fig 2). Ports where each species was observed at least once were more frequent in the west than in the east. For both the survey and presence-only datasets, ports with numerous observations coincide with ports that have a large volume of imported containers (e.g., TYO, YOK, and UKB). In addition, the species was observed over a wide area in the western part of Japan, where the industrial area is located. However, the number of observations was small in the ports at high latitudes.

The domestic container flow network during the analysis period exhibited hub-and-spoke system characteristics (Fig 1B). Two large clusters were detected: a group in eastern Japan centered on TYO and YOK, and a group in the west centered on UKB and OSK. Ports belonging to different clusters are rarely connected, and the connectivity matrix is sparse. The dependencies of the random variables were structured based on the connectivity.

### 3.2 Model comparison and introduction risk

This study aimed to determine whether the MCAR model improves the fit of the survey data. To test the effectiveness of the models, they were compared using DIC (Table 1). The joint model integrating presence-only data (MCAR-dt) for *S*. *invicta* slightly improved the DIC for survey data by 1.53 (60.08–58.55) compared to GLM and 1.1 (59.65–58.55) when compared to CAR. Similarly, MCAR-dt for *S*. *geminata* improved the DIC by 2.49 (70.11–67.62) compared to GLM and 1.91 (69.53–67.62) compared to CAR. These models also improved the fit of presence-only data, indicating that presence-only data may improve the prediction relative to a model that considers only survey data. The model integrating the survey data of the two species, MCAR-sp, also improved the prediction compared with the GLM and univariate CAR. The DIC of MCAR-sp improved the fit of the survey data of both species by 4.47 (130.19–125.72) compared to GLM and 3.46 (129.18–125.72) compared to CAR. Therefore, integrating the survey data of multiple species improved prediction from a single dataset. A comparison using WAIC also showed similar results (S4 Table in S1 File). The univariate CAR models were not effective for our dataset. The differences in DIC between the univariate CAR model and the GLM estimated from the survey data were less than 1 for both species.

**Table 1:** Estimated DIC values for each model. The survey and presence-only (PO) values for the joint model represent the local DIC, which is the DIC value for each dataset in the model.

Model type	Model	*S*. *invicta*			*S*. *geminata*			
		A	B	A+B	C	D	C+D	A+C
		Survey	PO	Total	Survey	PO	Total	Total
Separate	GLM	60.08	66.06	126.14	70.11	113.82	183.93	130.19
	CAR	59.65	65.89	125.54	69.53	107.1	176.63	129.18
Joint	MCAR-dt	58.55	65.64	124.19	67.62	100.22	167.84	-
	MCAR-sp	58.55	-	-	67.17	-	-	125.72

**Fig 3:**
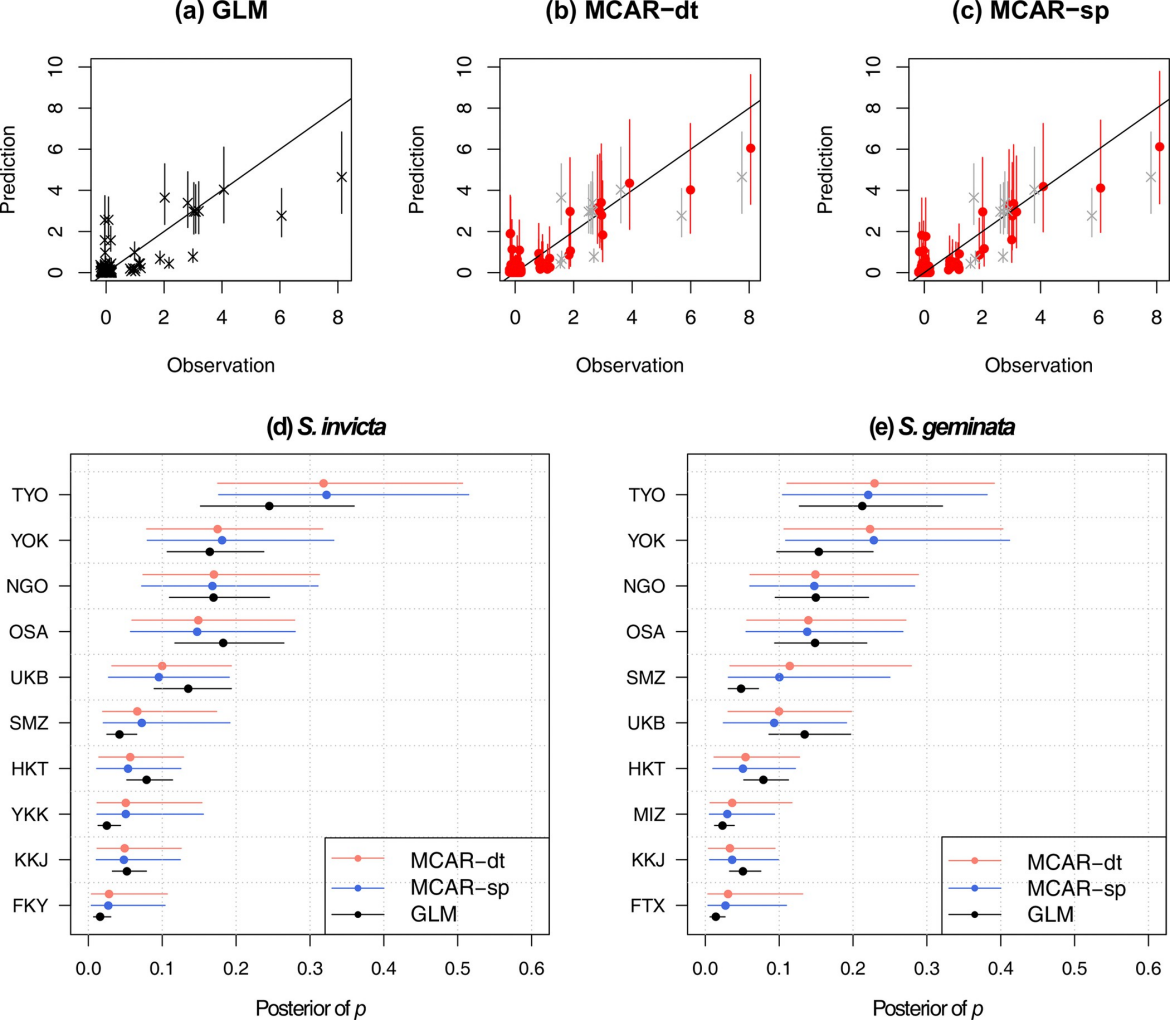
Prediction and posterior distribution. Comparison of prediction and observation estimated by the model (a) GLM, (b) MCAR-dt, and (c) MCAR-sp. The prediction shows the expected number of detections for each port, reflecting the estimated risk of introduction and the number of surveys conducted at each port, and bars show ranges based on the 95% credible interval. Results of the GLM with more than 2 observations are displayed in gray in (b) and (c). Posterior of *p*_*i*,*k*_ estimated for (d) *S*. *invicta* and (e) *S*. *geminata* using MCAR-dt (red), MCAR-sp (blue), and GLM (black). The ports are ordered by the results of the MCAR-dt, and only the 10-th high ranked ports are displayed.

The integrated models fit the observed data better, and the characteristics of the estimated introduction risk differed from those of the GLM. The GLM-predicted values diverged from the observed values, particularly in ports with a large number of observations (Fig 3A). However, in the MCAR models, the predictive mean was closer to the observations for large ports, and the prediction range was wider (Fig 3B and 3C). The estimated port priorities, which are of interest for practical applications, differed between the integration models and GLM (Fig 3D and 3E). Compared with the estimates using the GLM, the YOK was considered to have a higher relative risk for *S*. *geminata* based on the MCAR models. Although all models considered the main factor, the volume of imported containers, the GLM did not explain the observed survey data results, and the proposed integrated model provided a better fit.

### 3.3 Regression coefficient estimates

**Fig 4:**
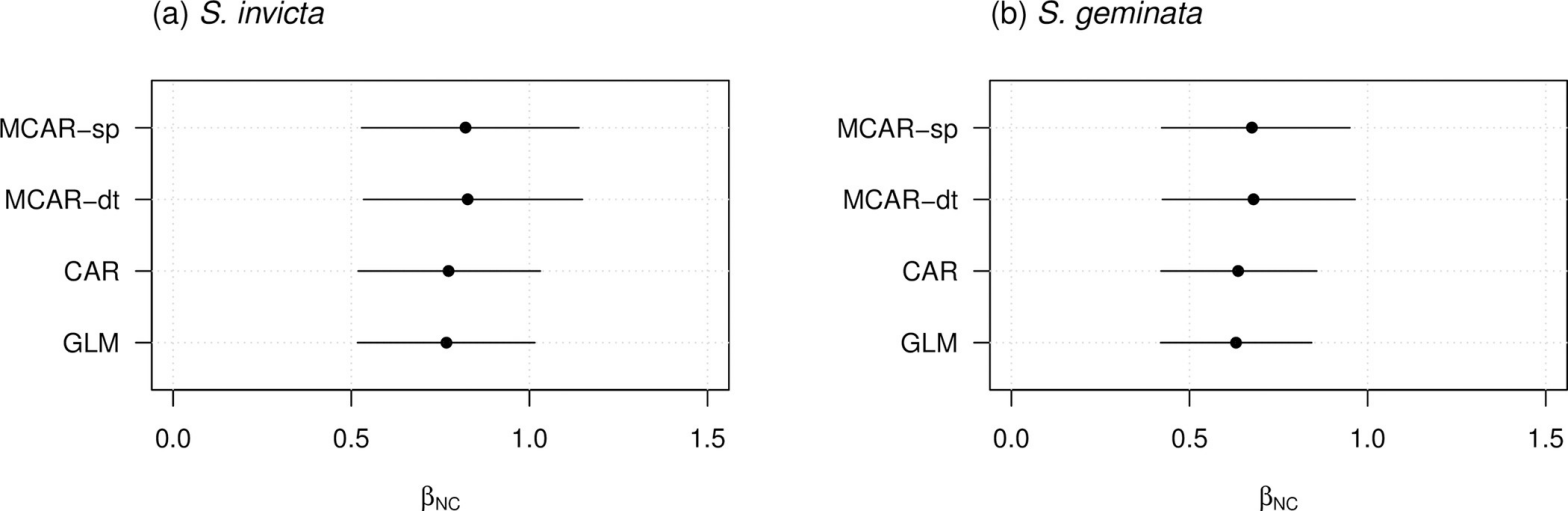
Posterior of fixed effect. Estimated posterior mean and the range between the 2.5th and 97.5th percentiles of the linear coefficient of the log-transformed number of containers (*β*_NC_) in each model for (a) *S*. *invicta* and (b) *S*. *geminata*.

We examined the posterior mean and 95% credible interval of the fixed effects for the models estimated from the survey data. The effect of the number of imported containers was significant positive in all models (Fig 4A and 4B). This result is consistent with previous reports that imported containers are the main factor in the introduction of alien species. Furthermore, in common with the two species, the posterior means of the joint models were larger than those of the GLM and univariate models, and the 95% credible intervals were larger in the positive direction. These results also suggest that the effect of NC, estimated using the univariate model, may have been underestimated.

In the process of selecting the explanatory variables, MT was excluded from all the models (S5 Table in S1 File). We confirmed that the 95% credible interval for the MT includes zero, indicating that the effect was not statistically significant in all models fitted to the survey data. However, this result does not necessarily indicate that the risk of introduction is independent of the climate. Although our data were processed without considering seasonal factors, the detection date on the presence-only data clearly suggested that seasonal variations had some effect on detection (S3 Table in S1 File).

### 3.4 Random effect estimates

The correlation coefficient between the dataset (*ρ*) assumed in the joint model was positive for MCAR-dt for *S*. *geminata* and MCAR-sp, with a posterior mean of 0.24 in the highest case (Table 2). The posterior of *ρ* in MCAR-dt for *S*. *invicta* did not indicate a positive correlation. These results coincide with those obtained with the model fit by DIC, indicating that the model improved when the information explaining the variance was transferred between the datasets. However, even in the models in which a positive correlation was found, no model was able to reject a correlation of zero from the 95% credit interval. Thus, the random effects of the network between the datasets may not have a large correlation.

**Table 2:** Mean and 95% credible interval (CI) of the posterior of the hyperparameters: (a) the correlation coefficient between data source *ρ* and the spatial dependence parameter *λ*, and (b) precision parameter *τ*.

(a)										
Model	Species	*ρ*					*λ*			
		Mean		95% CI			Mean	95% CI		
MCAR-dt	*S*. *invicta*	0.05		-0.54		0.62	0.44	0.06		0.87
MCAR-dt	*S*. *geminata*	0.24		-0.36		0.72	0.52	0.10		0.91
MCAR-sp	Joint	0.16		-0.47		0.71	0.41	0.04		0.88
(b)										
Model	*τ* (Survey)						*τ* (Presence-only)			
	Mean		95% CI				Mean	95% CI		
*S*. *invicta*										
CAR	103.38		5.75		410.73		108.05	6.70	422.64	
MCAR-dt	3.40		0.64		10.24		4.07	0.87	11.62	
MCAR-sp	3.33		0.64		10.08					
*S*. *geminata*										
CAR	101.83		5.55		405.43		3.62	0.75	11.93	
MCAR-dt	2.13		0.63		5.24		2.13	0.63	5.24	
MCAR-sp	3.09		0.58		9.58					

The posterior of the spatial dependence (λ) had a wide range and it showed little effective information. These results suggest that the dependence of the random variables on container logistics is not always large. The posterior of λ of MCAR-dt for *S*. *geminata* had a mean of slightly over 0.5 (Table 2). This parameter takes values between 0 and 1 and indicates the strength of the assumed dependence among the ports. Therefore, the results imply that the effect of the network in this model is slightly greater than that of the individual variability of each port.

The precision parameter (*τ*) of the random variable took a fairly large posterior mean in the univariate CAR models, indicating that the random effect was almost none (Table 2). However, *τ* of the MCAR model was relatively smaller than that of the univariate model, indicating that the estimation included variability modeled by CAR. This result is similar to the comparison of the model with DIC.

## 4. Discussion

A data integration model that considers a container logistics network was verified for the introduction of two fire ants, *S*. *invicta* and *S*. *geminata*, at Japanese ports. Some joint models improved the survey data prediction when compared with the GLM, without assuming the networks. These findings indicate that the joint modeling approach based on the network is practical for capturing the spread of species and addressing the lack of information in the early stages of introduction.

### 4.1 New insight by a joint model based on the network

The model provides the perspective that observations at one port would affect the risks at other ports that are strongly linked by logistics. Although a geographical spatial correlation could not generally be assumed in the introduction via container cargo at the Japanese scale, the logistics network had a hub-and-spoke structure rather than a random one. The assumption of a correlation using logistic connectivity seems reasonable because there have been many reports of cases in which containers have passed through multiple locations, which may influence the risk of introduction. The CAR considers the interaction between many points without a significant increase in the parameter dimensions. To the best of our knowledge, this is the first report to demonstrate the effectiveness of a new application of the CAR beyond the concept of space in invasion ecology. Moreover, considering the interaction between a large number of points would provide an opportunity to take new measures for ports usually managed independently by location.

The model provides two methods for effective data utilization in biological invasion. First, we demonstrate the effectiveness of integrating different data types, such as surveys and presence-only data. Pathway ports in Japan were investigated for presence-only data, in which contaminated containers were identified and aggregated with an introduction record. This additional information allowed the model to explain the variance in the survey data, especially for *S*. *geminata*, which was not only explained by the volume of the imported containers. Second, the effectiveness of integrating multispecies data that have a similar introduction process was demonstrated. The joint model improved the prediction of the survey data, because the two target ants had similar introduction and detection processes. This also emphasizes the importance of properly managing current observational data because it may help accurately estimate the risk of unknown invasive species that have not yet been introduced. This is one of the few examples demonstrating the effectiveness of data integration in the introduction of alien species.

### 4.2 Application and limitation

CAR models can be applied for invasive species management at ports. Port priority is crucial in biosecurity measures. The joint models that showed a better fit according to the DIC changed their priority based on the risk of introduction (Fig 3). In addition, although the random variables did not capture much of the variance in network connectivity, considering the networks in the statistical models is still effective. The Leroux CAR model applied in this study is effective in separately capturing individual port-specific variation and network dependence. Omitting variables, including those not observed, leads to an estimation bias [37]. Therefore, even if network effect detection is small, using a model such as CAR may be important for obtaining data on invasion by alien species.

Additionally, based on the results of the joint model for multiple species, the model developed in this study may be applicable outside our study area and outside ports. The model assumption made for a logistics network of containers is simple and can be easily replaced by a road or railway network [11]. The statistical model could also be extended to general species that have the same introduction process and cargo contamination in order to explain residuals that are not explained by existing species distribution models, regardless of geographical distance.

Statistical models allow us to analyze the effect of environmental variables on introduction risk, which is an advantage of statistical models. We did not find a significant effect of climate on the introduction risk. This may be because the objective of the model developed in this study was to determine the annual risk of introduction, and seasonality was not considered in data processing. This result may also be influenced by the differences between the grid-based climate data and the climate at the ports. It has been reported that the average temperature at which these species are established is higher than that in Japan, and that the potential locations of establishment estimated from the climate do not include Hokkaido, the northern part of Japan [25, 26]. However, *S*. *invicta* has been detected in trap surveys even at the port of Tomakomai (TMK), which is located in Hokkaido. The risk of biological invasion at ports should not be determined by the climate alone. Biosecurity strategy planners need to be aware of the difference between introduction and establishment, and validate each endpoint by analyzing the possible effects.

Our study has some limitations. Although we assumed that all ports were surveyed with the same effort in the port survey, the duration and number of traps may not have been completely standardized. Additionally, owing to the limitations of the statistical data used to determine the imported volume of containers, the models did not consider the transit of containers through the overseas ports. Typically, it is difficult to accurately identify the exchange of containers outside a country. This difficulty can be resolved using big data on vessel movements.

## 5. Conclusion

This study examined the effectiveness of a statistical model that considers the container logistics network and data integration using the network for the introduction of alien species via container cargo. The main finding was that the joint model using MCAR improved the prediction of the introduction risk and provided a method to address this lack of information. Beyond the concept of spatial characteristics, few studies have demonstrated the applicability of the CAR when considering networks for human-mediated species dispersion. These findings contribute to the development of more reliable strategies for controlling the introduction of alien species using statistical models.

## Supporting information

S1 File Supplementary tables, figure and additional information. Supplementary tables (S1–S5 Tables), figure (S1 Fig), and additional information for model implementation.
(DOCX)
