# Supplementary material for: Introduction risk of fire ants through container cargo in ports: Data integration approach considering a logistic network
Source: PLoS One. 2025 Feb 7;20(2):e0313849. doi: 10.1371/journal.pone.0313849 (PMC11805435; doi:10.1371/journal.pone.0313849)
Supplement: S1 File — Supplementary tables (S1–S5 Tables), figure (S1 Fig), and additional information for model implementation. (DOCX) [file pone.0313849.s001.docx]

**Supporting information**

Supplementary tables, figure and additional information.

Appendix S1: Supplementary tables and figure

Appendix S2: Supplement for model selection

Appendix S3: Supplement for model implementation

**Appendix S1: Supplementary tables and figures**

*Supplementary Tables:*

Table S1: List of analyzed ports. Port abbreviations are based on the United Nations Code for Trade and Transport Locations (UN/LOCODE).

| No. | Port | Abb. | No. | Port | Abb. | No. | Port | Abb. |
| --- | --- | --- | --- | --- | --- | --- | --- | --- |
| 1 | Muroran | MUR | 23 | Tsuruga | TRG | 45 | Tokushima_Komatsushima | KOM |
| 2 | Tomakomai | TMK | 24 | Shimizu | SMZ | 46 | Takamatsu | TAK |
| 3 | Ishikariwan_Shin | ICW | 25 | Omaezaki | OMZ | 47 | Matsuyama | MYJ |
| 4 | Hakodate | HKD | 26 | Nagoya | NGO | 48 | Mishima_Kawanoe | MKX |
| 5 | Otaru | OTR | 27 | Mikawa | MKW | 49 | Imabari | IMB |
| 6 | Kushiro | KUH | 28 | Yokkaichi | YKK | 50 | Kochi | KCZ |
| 7 | Hachinohe | HHE | 29 | Maizuru | MAI | 51 | Kitakyushu | KKJ |
| 8 | Kamaishi | KIS | 30 | Osaka | OSA | 52 | Hakata | HKT |
| 9 | Sendai_Shiokama | SGM | 31 | Sakai_senboku | SBK | 53 | Miike | MII |
| 10 | Akita | AXT | 32 | Kobe | UKB | 54 | Imari | IMI |
| 11 | Sakata | SKT | 33 | Wakayama_Shimotsu | SMT | 55 | Nagasaki | NGS |
| 12 | Onahama | ONA | 34 | Sakai | SMN | 56 | Yatsushiro | YAT |
| 13 | Ibaraki | HIC | 35 | Hamada | HMD | 57 | Kumamoto | KMJ |
| 14 | Kashima | KSM | 36 | Mizushima | MIZ | 58 | Oita | OIT |
| 15 | Chiba | CHB | 37 | Hiroshima | HIJ | 59 | Hososhima | HSM |
| 16 | Tokyo | TYO | 38 | Fukuyama | FKY | 60 | Aburatsu | ABU |
| 17 | Yokohama | YOK | 39 | Otake | OTK | 61 | Kagoshima | KOJ |
| 18 | Kawasaki | KWS | 40 | Shimonoseki | SHS | 62 | Shibushi | SBS |
| 19 | Niigata | KIJ | 41 | Tokuyama_Kudamatsu | TXD | 63 | Sendai | SEN |
| 20 | Naoetsu | NAO | 42 | Iwakuni | IWK | 64 | Naha | NAH |
| 21 | Fushiki_Toyama | FTX | 43 | Mitajiri_Nakanoseki | MJR | 65 | Ishigaki | ISG |
| 22 | Kanazawa | KNZ | 44 | Ube | UBJ |  |  |  |

Table S2 Aggregated result of port survey for (a) *S. invicta* and (b) *S. geminata* (1=Detect; 2=Not detect; N=Not surveyed). The “Port” column corresponds to the numbers listed in Table S1.

(a) *S. invicta*

| **Port** | **17_0** | **17_1** | **17_2** | **17_3** | **17_4** | **18_1** | **18_2** | **19_1** | **19_2** | **19_3** | **20_1** | **20_2** | **21_1** | **21_2** | **22_1** | **22_2** | **22_3** | **22_4** | **22_5** | **22_6** | **22_7** | **22_8** |
| --- | --- | --- | --- | --- | --- | --- | --- | --- | --- | --- | --- | --- | --- | --- | --- | --- | --- | --- | --- | --- | --- | --- |
| 1 | N | 0 | 0 | 0 | N | 0 | 0 | 0 | 0 | N | 0 | 0 | 0 | 0 | N | N | N | N | N | N | 0 | 0 |
| 2 | N | 0 | 0 | 0 | N | 1 | 0 | 0 | 0 | N | 0 | 0 | 0 | 0 | N | 0 | 0 | 0 | 0 | N | N | N |
| 3 | N | 0 | 0 | 0 | N | 0 | 0 | 0 | 0 | 0 | 0 | 0 | 0 | 0 | N | N | N | N | N | N | 0 | 0 |
| 4 | N | 0 | 0 | 0 | N | 0 | 0 | 0 | 0 | N | 0 | 0 | 0 | 0 | N | N | N | N | N | N | 0 | 0 |
| 5 | N | 0 | 0 | 0 | N | 0 | 0 | 0 | 0 | 0 | 0 | 0 | 0 | 0 | N | N | N | N | N | N | 0 | 0 |
| 6 | N | 0 | 0 | 0 | N | 0 | 0 | 0 | 0 | 0 | 0 | 0 | 0 | 0 | N | N | N | N | N | N | 0 | 0 |
| 7 | N | 0 | 0 | 0 | N | 0 | 0 | 0 | 0 | 0 | 0 | 0 | 0 | 0 | N | N | N | N | N | N | 0 | 0 |
| 8 | N | 0 | 0 | 0 | N | 0 | 0 | 0 | 0 | 0 | 0 | 0 | 0 | 0 | N | N | N | N | N | N | 0 | 0 |
| 9 | N | 0 | 0 | 0 | N | 0 | 0 | 0 | 0 | 0 | 0 | 0 | 0 | 0 | N | N | N | N | N | N | 0 | 0 |
| 10 | N | 0 | 0 | 0 | N | 0 | 0 | 0 | 0 | 0 | 0 | 0 | 0 | 0 | N | N | N | N | N | N | 0 | 0 |
| 11 | N | 0 | 0 | 0 | N | 0 | 0 | 0 | 0 | 0 | 0 | 0 | 0 | 0 | N | N | N | N | N | N | 0 | 0 |
| 12 | N | 0 | 0 | 0 | N | 0 | 0 | 0 | 0 | 0 | 0 | 0 | 0 | 0 | N | N | N | N | N | N | 0 | 0 |
| 13 | N | 0 | 0 | 0 | N | 0 | 0 | 0 | 0 | N | 0 | 0 | 0 | 0 | N | N | N | N | N | N | 0 | 0 |
| 14 | N | 0 | 0 | 0 | N | 0 | 0 | 0 | 0 | N | 0 | 0 | 0 | 0 | N | N | N | N | N | N | 0 | 0 |
| 15 | N | 0 | 0 | 0 | N | 0 | 0 | 0 | 0 | 0 | 0 | 1 | 0 | 0 | 0 | 0 | N | 0 | 0 | N | N | N |
| 16 | 0 | 0 | 0 | 0 | 0 | 0 | 0 | 1 | 1 | 1 | 1 | 1 | 1 | 1 | 0 | 1 | N | 0 | 0 | 0 | N | N |
| 17 | 1 | 0 | 0 | 0 | 0 | 0 | 0 | 0 | 0 | 0 | 1 | 1 | 0 | 0 | 0 | 0 | N | 0 | 0 | N | N | N |
| 18 | N | 0 | 0 | 0 | N | 0 | 0 | 0 | 0 | 0 | 0 | 0 | 0 | 0 | N | N | N | N | N | N | 0 | 0 |
| 19 | N | 0 | 0 | 0 | N | 0 | 0 | 0 | 0 | 0 | 0 | 0 | 0 | 0 | 0 | 0 | N | 0 | 0 | N | N | N |
| 20 | N | 0 | 0 | 0 | N | 0 | 0 | 0 | 0 | 0 | 0 | 0 | 0 | 0 | N | N | N | N | N | N | 0 | 0 |
| 21 | N | 0 | 0 | 0 | N | 0 | 0 | 0 | 0 | 0 | 0 | 0 | 0 | 0 | N | N | N | N | N | N | 0 | 0 |
| 22 | N | 0 | 0 | 0 | N | 0 | 0 | 0 | 0 | 0 | 0 | 0 | 0 | 0 | N | N | N | N | N | N | 0 | 0 |
| 23 | N | 0 | 0 | 0 | N | 0 | 0 | 0 | 0 | 0 | 0 | 0 | 0 | 0 | N | N | N | N | N | N | 0 | 0 |
| 24 | N | 1 | 0 | 0 | 0 | 1 | 0 | 0 | 0 | N | 0 | 0 | 0 | 0 | 0 | 0 | N | 0 | 0 | N | N | N |
| 25 | N | 0 | 0 | 0 | N | 0 | 0 | 0 | 0 | N | 0 | 0 | 0 | 0 | N | N | N | N | N | N | 0 | 0 |
| 26 | 0 | 0 | 1 | 0 | 0 | 0 | 0 | 0 | 0 | 0 | 0 | 1 | 0 | 1 | 0 | 0 | 0 | 0 | 0 | 0 | N | N |
| 27 | N | 0 | 0 | 0 | N | 0 | 0 | 0 | 0 | 0 | 0 | 0 | 0 | 0 | N | N | N | N | N | N | 0 | 0 |
| 28 | N | 0 | 0 | 0 | N | 0 | 0 | 0 | 0 | 1 | 0 | 0 | 0 | 1 | 0 | 0 | N | 0 | 0 | 0 | N | N |
| 29 | N | 0 | 0 | 0 | N | 0 | 0 | 0 | 0 | N | 0 | 0 | 0 | 0 | N | N | N | N | N | N | 0 | 0 |
| 30 | 0 | 0 | 0 | 0 | 0 | 0 | 0 | 0 | 0 | 0 | 1 | 0 | 1 | 0 | 0 | 0 | 0 | 0 | 0 | 0 | N | N |
| 31 | N | 0 | 0 | 0 | N | 0 | 0 | 0 | 0 | 0 | 0 | 0 | 0 | 0 | N | N | N | N | N | N | 0 | 0 |
| 32 | 0 | 0 | 0 | 0 | 0 | 0 | 0 | 0 | 0 | N | 0 | 0 | 0 | 0 | 0 | 0 | 0 | 0 | 0 | 0 | N | N |
| 33 | N | 0 | 0 | 0 | N | 0 | 0 | 0 | 0 | 0 | 0 | 0 | 0 | 0 | N | N | N | N | N | N | 0 | 0 |
| 34 | N | 0 | 0 | 0 | N | 0 | 0 | 0 | 0 | 0 | 0 | 0 | 0 | 0 | N | N | N | N | N | N | 0 | 0 |
| 35 | N | 0 | 0 | 0 | N | 0 | 0 | 0 | 0 | 0 | 0 | 0 | 0 | 0 | N | N | N | N | N | N | 0 | 0 |
| 36 | N | 0 | 0 | 0 | 0 | 0 | 0 | 0 | 0 | 0 | 0 | 0 | 0 | 0 | 0 | 0 | 0 | 0 | 0 | 0 | N | N |
| 37 | N | 1 | 0 | 0 | 0 | 0 | 0 | 0 | 0 | 0 | 0 | 0 | 0 | 0 | 0 | 0 | 0 | 0 | 0 | 0 | N | N |
| 38 | N | 0 | 0 | 0 | N | 0 | 0 | 0 | 0 | 0 | 0 | 0 | 0 | 0 | N | N | N | N | N | N | 0 | 1 |
| 39 | N | 0 | 0 | 0 | N | 0 | 0 | 0 | 0 | 0 | 0 | 0 | 0 | 0 | N | N | N | N | N | N | 0 | 0 |
| 40 | N | 0 | 0 | 0 | N | 0 | 0 | 0 | 0 | 0 | 0 | 0 | 0 | 0 | N | N | N | N | N | N | 0 | 0 |
| 41 | N | 0 | 0 | 0 | N | 0 | 0 | 0 | 0 | 0 | 0 | 0 | 0 | 0 | N | N | N | N | N | N | N | 0 |
| 42 | N | 0 | 0 | 0 | N | 0 | 0 | 0 | 0 | 0 | 0 | 0 | 0 | 0 | N | N | N | N | N | N | 0 | 0 |
| 43 | N | 0 | 0 | 0 | N | 0 | 0 | 0 | 0 | 0 | 0 | 0 | 0 | 0 | N | N | N | N | N | N | 0 | 0 |
| 44 | N | 0 | 0 | 0 | N | 0 | 0 | 0 | 0 | 0 | 0 | 0 | 0 | 0 | N | N | N | N | N | N | 0 | 0 |
| 45 | N | 0 | 0 | 0 | N | 0 | 0 | 0 | 0 | 0 | 0 | 0 | 0 | 0 | N | N | N | N | N | N | 0 | 0 |
| 46 | N | 0 | 0 | 0 | N | 0 | 0 | 0 | 0 | 0 | 0 | 0 | 0 | 0 | N | N | N | N | N | N | 0 | 0 |
| 47 | N | 0 | 0 | 0 | N | 0 | 0 | 0 | 0 | 0 | 0 | 0 | 0 | 0 | N | N | N | N | N | N | 0 | 0 |
| 48 | N | 0 | 0 | 0 | N | 0 | 0 | 0 | 0 | 0 | 0 | 0 | 0 | 0 | N | N | N | N | N | N | 0 | 0 |
| 49 | N | 0 | 0 | 0 | N | 0 | 0 | 0 | 0 | 0 | 0 | 0 | 0 | 0 | N | N | N | N | N | N | 0 | 0 |
| 50 | N | 0 | 0 | 0 | N | 0 | 0 | 0 | 0 | 0 | 0 | 0 | 0 | 0 | N | N | N | N | N | N | 0 | 0 |
| 51 | N | 0 | 1 | 0 | 0 | 0 | 0 | 0 | 0 | 0 | 0 | 0 | 0 | 0 | 0 | 0 | N | 0 | 0 | N | 0 | 0 |
| 52 | 0 | 0 | 0 | 0 | 0 | 0 | 0 | 0 | 0 | 0 | 0 | 0 | 0 | 0 | 0 | 0 | N | 0 | N | 0 | 0 | 0 |
| 53 | N | 0 | 0 | 0 | N | 0 | 0 | 0 | 0 | 0 | 0 | 0 | 0 | 0 | N | N | N | N | N | N | 0 | 0 |
| 54 | N | 0 | 0 | 0 | N | 0 | 0 | 0 | 0 | 0 | 0 | 0 | 0 | 0 | N | N | N | N | N | N | 0 | 0 |
| 55 | N | 0 | 0 | 0 | N | 0 | 0 | 0 | 0 | 0 | 0 | 0 | 0 | 0 | N | N | N | N | N | N | 0 | 0 |
| 56 | N | 0 | 0 | 0 | N | 0 | 0 | 0 | 0 | 0 | 0 | 0 | 0 | 0 | N | N | N | N | N | N | 0 | 0 |
| 57 | N | 0 | 0 | 0 | N | 0 | 0 | 0 | 0 | 0 | 0 | 0 | 0 | 0 | N | N | N | N | N | N | 0 | 0 |
| 58 | N | 0 | 0 | 0 | N | 0 | 0 | 0 | 0 | 0 | 0 | 0 | 0 | 0 | N | N | N | N | N | N | 0 | 0 |
| 59 | N | 0 | 0 | 0 | N | 0 | 0 | 0 | 0 | 0 | 0 | 0 | 0 | 0 | N | N | N | N | N | N | 0 | 0 |
| 60 | N | 0 | 0 | 0 | N | 0 | 0 | 0 | 0 | 0 | 0 | 0 | 0 | 0 | N | N | N | N | N | N | 0 | 0 |
| 61 | N | 0 | 0 | 0 | N | 0 | 0 | 0 | 0 | 0 | 0 | 0 | 0 | 0 | N | N | N | N | N | N | 0 | 0 |
| 62 | N | 0 | 0 | 0 | N | 0 | 0 | 0 | 0 | N | 0 | 0 | 0 | 0 | N | N | N | N | N | N | 0 | 0 |
| 63 | N | 0 | 0 | 0 | N | 0 | 0 | 0 | 0 | 0 | 0 | 0 | 0 | 0 | N | N | N | N | N | N | 0 | 0 |
| 64 | 0 | 0 | 0 | 0 | 0 | 0 | 0 | 0 | 0 | N | 0 | 0 | 0 | 0 | N | N | N | N | N | N | 0 | 0 |
| 65 | N | 0 | 0 | 0 | N | 0 | 0 | 0 | 0 | 0 | 0 | 0 | 0 | 0 | N | N | N | N | N | N | 0 | 0 |

(b) *S. geminata*

| **Port** | **17_0** | **17_1** | **17_2** | **17_3** | **17_4** | **18_1** | **18_2** | **19_1** | **19_2** | **19_3** | **20_1** | **20_2** | **21_1** | **21_2** | **22_1** | **22_2** | **22_3** | **22_4** | **22_5** | **22_6** | **22_7** | **22_8** |
| --- | --- | --- | --- | --- | --- | --- | --- | --- | --- | --- | --- | --- | --- | --- | --- | --- | --- | --- | --- | --- | --- | --- |
| 1 | N | 0 | 0 | 0 | N | 0 | 0 | 0 | 0 | N | 0 | 0 | 0 | 0 | N | N | N | N | N | N | 0 | 0 |
| 2 | N | 0 | 0 | 0 | N | 0 | 0 | 0 | 0 | N | 0 | 0 | 0 | 0 | N | 0 | 0 | 0 | 0 | N | N | N |
| 3 | N | 0 | 0 | 0 | N | 0 | 0 | 0 | 0 | 0 | 0 | 0 | 0 | 0 | N | N | N | N | N | N | 0 | 0 |
| 4 | N | 0 | 0 | 0 | N | 0 | 0 | 0 | 0 | N | 0 | 0 | 0 | 0 | N | N | N | N | N | N | 0 | 0 |
| 5 | N | 0 | 0 | 0 | N | 0 | 0 | 0 | 0 | 0 | 0 | 0 | 0 | 0 | N | N | N | N | N | N | 0 | 0 |
| 6 | N | 0 | 0 | 0 | N | 0 | 0 | 0 | 0 | 0 | 0 | 0 | 0 | 0 | N | N | N | N | N | N | 0 | 0 |
| 7 | N | 0 | 0 | 0 | N | 0 | 0 | 0 | 0 | 0 | 0 | 0 | 0 | 0 | N | N | N | N | N | N | 0 | 0 |
| 8 | N | 0 | 0 | 0 | N | 0 | 0 | 0 | 0 | 0 | 0 | 0 | 0 | 0 | N | N | N | N | N | N | 0 | 0 |
| 9 | N | 0 | 0 | 0 | N | 0 | 0 | 0 | 0 | 0 | 0 | 0 | 0 | 0 | N | N | N | N | N | N | 0 | 0 |
| 10 | N | 0 | 0 | 0 | N | 0 | 0 | 0 | 0 | 0 | 0 | 0 | 0 | 0 | N | N | N | N | N | N | 0 | 0 |
| 11 | N | 0 | 0 | 0 | N | 0 | 0 | 0 | 0 | 0 | 0 | 0 | 0 | 0 | N | N | N | N | N | N | 0 | 0 |
| 12 | N | 0 | 0 | 0 | N | 0 | 0 | 0 | 0 | 0 | 0 | 0 | 0 | 0 | N | N | N | N | N | N | 0 | 0 |
| 13 | N | 0 | 0 | 0 | N | 0 | 0 | 0 | 0 | N | 0 | 0 | 0 | 0 | N | N | N | N | N | N | 0 | 0 |
| 14 | N | 0 | 0 | 0 | N | 0 | 0 | 0 | 0 | N | 0 | 0 | 0 | 0 | N | N | N | N | N | N | 0 | 0 |
| 15 | N | 0 | 0 | 0 | N | 0 | 0 | 0 | 0 | 0 | 0 | 0 | 0 | 0 | 0 | 0 | N | 0 | 0 | N | N | N |
| 16 | 1 | 0 | 0 | 0 | 0 | 0 | 0 | 0 | 1 | 0 | 0 | 0 | 1 | 0 | 0 | 1 | N | 0 | 0 | 0 | N | N |
| 17 | 0 | 0 | 0 | 0 | 0 | 0 | 0 | 0 | 1 | 1 | 1 | 1 | 1 | 0 | 0 | 1 | N | 0 | 0 | N | N | N |
| 18 | N | 0 | 0 | 0 | N | 0 | 0 | 0 | 0 | 0 | 0 | 0 | 0 | 0 | N | N | N | N | N | N | 0 | 0 |
| 19 | N | 0 | 0 | 0 | N | 0 | 0 | 0 | 0 | 0 | 0 | 0 | 0 | 0 | 0 | 0 | N | 0 | 0 | N | N | N |
| 20 | N | 0 | 0 | 0 | N | 0 | 0 | 0 | 0 | 0 | 0 | 0 | 0 | 0 | N | N | N | N | N | N | 0 | 0 |
| 21 | N | 0 | 0 | 0 | N | 0 | 0 | 0 | 1 | 0 | 0 | 0 | 0 | 0 | N | N | N | N | N | N | 0 | 0 |
| 22 | N | 0 | 0 | 0 | N | 0 | 0 | 0 | 0 | 0 | 0 | 0 | 0 | 0 | N | N | N | N | N | N | 0 | 0 |
| 23 | N | 0 | 0 | 0 | N | 0 | 0 | 0 | 0 | 0 | 0 | 0 | 0 | 0 | N | N | N | N | N | N | 0 | 0 |
| 24 | N | 1 | 0 | 0 | 0 | 0 | 0 | 0 | 1 | N | 0 | 0 | 1 | 0 | 0 | 0 | N | 0 | 0 | N | N | N |
| 25 | N | 0 | 0 | 0 | N | 0 | 0 | 0 | 0 | N | 0 | 1 | 0 | 0 | N | N | N | N | N | N | 0 | 0 |
| 26 | 0 | 0 | 1 | 0 | 0 | 0 | 0 | 0 | 0 | 0 | 0 | 0 | 0 | 0 | 0 | 1 | 0 | 1 | 0 | 0 | N | N |
| 27 | N | 0 | 0 | 0 | N | 0 | 0 | 0 | 0 | 0 | 0 | 0 | 0 | 0 | N | N | N | N | N | N | 0 | 0 |
| 28 | N | 0 | 0 | 0 | N | 0 | 0 | 0 | 0 | 0 | 0 | 0 | 0 | 0 | 0 | 0 | N | 0 | 0 | 0 | N | N |
| 29 | N | 0 | 0 | 0 | N | 0 | 0 | 0 | 0 | N | 0 | 0 | 0 | 0 | N | N | N | N | N | N | 0 | 0 |
| 30 | 0 | 0 | 0 | 0 | 0 | 0 | 0 | 0 | 0 | 0 | 1 | 0 | 1 | 1 | 0 | 0 | 0 | 0 | 0 | 0 | N | N |
| 31 | N | 0 | 0 | 0 | N | 0 | 0 | 0 | 0 | 0 | 0 | 0 | 0 | 0 | N | N | N | N | N | N | 0 | 0 |
| 32 | 0 | 0 | 0 | 0 | 0 | 0 | 0 | 0 | 0 | N | 0 | 0 | 0 | 0 | 0 | 0 | 0 | 0 | 0 | 0 | N | N |
| 33 | N | 0 | 0 | 0 | N | 0 | 0 | 0 | 0 | 0 | 0 | 0 | 0 | 0 | N | N | N | N | N | N | 0 | 0 |
| 34 | N | 0 | 0 | 0 | N | 0 | 0 | 0 | 0 | 0 | 0 | 0 | 0 | 0 | N | N | N | N | N | N | 0 | 0 |
| 35 | N | 0 | 0 | 0 | N | 0 | 0 | 0 | 0 | 0 | 0 | 0 | 0 | 0 | N | N | N | N | N | N | 0 | 0 |
| 36 | N | 0 | 0 | 0 | 0 | 0 | 0 | 0 | 0 | 0 | 0 | 0 | 0 | 0 | 0 | 0 | 0 | 1 | 0 | 0 | N | N |
| 37 | N | 0 | 0 | 0 | 0 | 0 | 0 | 0 | 0 | 0 | 0 | 0 | 0 | 0 | 0 | 1 | 0 | 0 | 0 | 0 | N | N |
| 38 | N | 0 | 0 | 0 | N | 0 | 0 | 0 | 0 | 0 | 0 | 0 | 0 | 0 | N | N | N | N | N | N | 0 | 0 |
| 39 | N | 0 | 0 | 0 | N | 0 | 0 | 0 | 0 | 0 | 0 | 0 | 0 | 0 | N | N | N | N | N | N | 0 | 0 |
| 40 | N | 0 | 0 | 0 | N | 0 | 0 | 0 | 0 | 0 | 0 | 0 | 0 | 0 | N | N | N | N | N | N | 0 | 0 |
| 41 | N | 0 | 0 | 0 | N | 0 | 0 | 0 | 0 | 0 | 0 | 0 | 0 | 0 | N | N | N | N | N | N | N | 0 |
| 42 | N | 0 | 0 | 0 | N | 0 | 0 | 0 | 0 | 0 | 0 | 0 | 0 | 0 | N | N | N | N | N | N | 0 | 0 |
| 43 | N | 0 | 0 | 0 | N | 0 | 0 | 0 | 0 | 0 | 0 | 0 | 0 | 0 | N | N | N | N | N | N | 0 | 0 |
| 44 | N | 0 | 0 | 0 | N | 0 | 0 | 0 | 0 | 0 | 0 | 0 | 0 | 0 | N | N | N | N | N | N | 0 | 0 |
| 45 | N | 0 | 0 | 0 | N | 0 | 0 | 0 | 0 | 0 | 0 | 0 | 0 | 0 | N | N | N | N | N | N | 0 | 0 |
| 46 | N | 0 | 0 | 0 | N | 0 | 0 | 0 | 0 | 0 | 0 | 0 | 0 | 0 | N | N | N | N | N | N | 0 | 0 |
| 47 | N | 0 | 0 | 0 | N | 0 | 0 | 0 | 0 | 0 | 1 | 0 | 0 | 0 | N | N | N | N | N | N | 0 | 0 |
| 48 | N | 0 | 0 | 0 | N | 0 | 0 | 0 | 0 | 0 | 0 | 0 | 0 | 0 | N | N | N | N | N | N | 0 | 0 |
| 49 | N | 0 | 0 | 0 | N | 0 | 0 | 0 | 0 | 0 | 0 | 0 | 0 | 0 | N | N | N | N | N | N | 0 | 0 |
| 50 | N | 0 | 0 | 0 | N | 0 | 0 | 0 | 0 | 0 | 0 | 0 | 0 | 0 | N | N | N | N | N | N | 0 | 0 |
| 51 | N | 0 | 0 | 0 | 0 | 0 | 0 | 0 | 0 | 0 | 0 | 0 | 0 | 0 | 0 | 0 | N | 0 | 0 | N | 0 | 0 |
| 52 | 0 | 0 | 0 | 0 | 0 | 0 | 0 | 0 | 0 | 0 | 0 | 0 | 0 | 0 | 0 | 0 | N | 0 | N | 0 | 0 | 0 |
| 53 | N | 0 | 0 | 0 | N | 0 | 0 | 0 | 0 | 0 | 0 | 0 | 0 | 0 | N | N | N | N | N | N | 0 | 0 |
| 54 | N | 0 | 0 | 0 | N | 0 | 0 | 0 | 0 | 0 | 0 | 0 | 0 | 0 | N | N | N | N | N | N | 0 | 0 |
| 55 | N | 0 | 0 | 0 | N | 0 | 0 | 0 | 0 | 0 | 0 | 0 | 0 | 0 | N | N | N | N | N | N | 0 | 0 |
| 56 | N | 0 | 0 | 0 | N | 0 | 0 | 0 | 0 | 0 | 0 | 0 | 0 | 0 | N | N | N | N | N | N | 0 | 0 |
| 57 | N | 0 | 0 | 0 | N | 0 | 0 | 0 | 0 | 0 | 0 | 0 | 0 | 0 | N | N | N | N | N | N | 0 | 0 |
| 58 | N | 0 | 0 | 0 | N | 0 | 0 | 0 | 0 | 0 | 0 | 0 | 0 | 0 | N | N | N | N | N | N | 0 | 0 |
| 59 | N | 0 | 0 | 0 | N | 0 | 0 | 0 | 0 | 0 | 0 | 0 | 0 | 0 | N | N | N | N | N | N | 0 | 0 |
| 60 | N | 0 | 0 | 0 | N | 0 | 0 | 0 | 0 | 0 | 0 | 0 | 0 | 0 | N | N | N | N | N | N | 0 | 0 |
| 61 | N | 0 | 0 | 0 | N | 0 | 0 | 0 | 0 | 0 | 0 | 0 | 0 | 0 | N | N | N | N | N | N | 0 | 0 |
| 62 | N | 0 | 0 | 0 | N | 0 | 0 | 0 | 0 | N | 0 | 0 | 0 | 0 | N | N | N | N | N | N | 0 | 1 |
| 63 | N | 0 | 0 | 0 | N | 0 | 0 | 0 | 0 | 0 | 0 | 0 | 0 | 0 | N | N | N | N | N | N | 0 | 0 |
| 64 | 0 | 0 | 0 | 0 | 0 | 0 | 0 | 0 | 0 | N | 0 | 0 | 0 | 0 | N | N | N | N | N | N | 0 | 0 |
| 65 | N | 0 | 0 | 0 | N | 0 | 0 | 0 | 0 | 0 | 0 | 0 | 0 | 0 | N | N | N | N | N | N | 0 | 0 |

Table S3 List of introduction records of (a) *S. invicta* and (b) *S. geminata* from 2017 to 2022 (excluding detection from nationwide port surveys). Among the available introduction records, only those in which the port of entries could be identified were included. Records were obtained from a website publicly provided by the Ministry of the Environment, Japan (<https://www.env.go.jp/nature/intro/2outline/attention/hiari.html>, in Japanese).

(a) *S. invicta*

| Announced | Port | Announced | Port |
| --- | --- | --- | --- |
| 2017/06/13 | UKB | 2019/02/18 | OSA |
| 2017/06/18 | UKB | 2019/06/18 | NGO |
| 2017/06/30 | NGO | 2019/07/05 | TYO |
| 2017/07/04 | OSA | 2019/07/18 | OSA |
| 2017/07/06 | TYO | 2019/09/10 | YOK |
| 2017/07/10 | NGO | 2019/09/11 | YOK |
| 2017/07/21 | HKT | 2019/10/21 | TYO |
| 2017/07/25 | KKJ | 2020/06/11 | TYO |
| 2017/07/27 | HKT | 2020/06/19 | YOK |
| 2017/08/04 | NGO | 2020/06/23 | TYO |
| 2017/08/09 | MIZ | 2020/06/24 | CHB |
| 2017/08/17 | TYO | 2020/07/03 | YOK |
| 2017/09/01 | NGO | 2020/09/25 | NGO |
| 2017/09/06 | YOK | 2020/10/23 | TYO |
| 2017/10/03 | UKB | 2021/05/25 | YOK |
| 2017/10/14 | YOK | 2021/06/21 | TYO |
| 2017/11/06 | OSA | 2021/08/12 | TYO |
| 2017/11/09 | NGO | 2021/08/18 | NGO |
| 2017/11/22 | HIJ | 2021/08/23 | TYO |
| 2017/11/22 | HIJ | 2021/08/27 | HKT |
| 2018/05/10 | HIJ | 2021/09/28 | OSA |
| 2018/06/15 | OSA | 2021/09/13 | TYO |
| 2018/06/16 | OSA | 2022/06/28 | TYO |
| 2018/07/05 | OSA | 2022/07/08 | NGO |
| 2018/07/20 | NGO | 2022/07/28 | HKT |
| 2018/07/31 | NGO | 2022/11/02 | TYO |
| 2018/08/23 | HIJ | 2022/11/03 | FKY |
| 2018/08/29 | NGO |  |  |

(b) *S. geminata*

Obtained website (<https://www.env.go.jp/nature/intro/2outline/attention/02_general/file/akakamiari_ichiran.pdf>, in Japanese)

| Confirmed | Port of entry | Confirmed | Port of entry |
| --- | --- | --- | --- |
| 2017/06/20 | UKB | 2019/06/27 | HKT |
| 2017/06/23 | OSA | 2019/07/19 | UKB |
| 2017/06/23 | OSA | 2019/10/05 | NGO |
| 2017/07/09 | NGO | 2019/12/26 | HKT |
| 2017/07/12 | TYO | 2020/01/10 | HKT |
| 2017/07/24 | MKX | 2020/06/26 | TYO |
| 2017/08/02 | NGO | 2020/07/10 | TYO |
| 2017/07/28 | SMZ | 2020/07/15 | MYJ (MKX, IMB) |
| 2017/08/10 | MNX | 2020/07/30 | YOK |
| 2017/08/13 | TYO | 2020/09/23 | UKB |
| 2017/08/24 | SMZ | 2020/10/04 | MIZ |
| 2017/09/06 | SMZ | 2020/10/06 | YOK |
| 2017/09/08 | SMZ | 2020/10/16 | YOK |
| 2017/09/28 | KKJ | 2020/12/08 | TYO |
| 2017/10/04 | YOK (TYO) | 2021/06/17 | KKJ |
| 2018/04/26 | UKB | 2021/07/26 | OSA |
| 2018/05/31 | UKB | 2021/08/18 | TYO |
| 2018/05/30 | TYO | 2021/08/23 | TYO |
| 2018/06/22 | TYO | 2021/08/26 | NGO |
| 2018/07/02 | TYO | 2021/10/08 | TYO |
| 2018/07/04 | SMZ | 2021/10/27 | TYO |
| 2018/07/06 | OSA | 2021/11/15 | TYO |
| 2018/07/18 | SMZ | 2022/08/01 | TYO |
| 2018/08/27 | UKB | 2022/08/16 | TYO |
| 2018/09/17 | MIZ | 2022/08/22 | TYO |
| 2018/10/24 | YOK | 2022/08/30 | TYO |
| 2018/11/13 | SMZ | 2022/10/07 | HKT |
| 2019/04/05 | SMZ | 2022/12/12 | TYO |
| 2019/06/09 | MIZ |  |  |

*Note:* Parentheses indicate the identified domestic ports of transit or ports of call after loading.

Table S4 Results of WAIC shown in a similar way as Table 1 in the manuscript.

| Model type | Model | *S. invicta* | | |  | *S. geminata* | | |  |
| --- | --- | --- | --- | --- | --- | --- | --- | --- | --- |
|  |  | A | B | A+B |  | C | D | C+D | A+C |
|  |  | Survey | PO | Total |  | Survey | PO | Total | Total |
| Separate | GLM | 61.00 | 66.49 | 127.49 |  | 70.76 | 117.51 | 188.27 | 131.76 |
|  | CAR | 60.69 | 66.31 | 127.00 |  | 70.47 | 126.64 | 197.11 | 131.16 |
| Joint | MCAR-dt | 58.73 | 66.9 | 125.63 |  | 68.19 | 104.42 | 172.61 | - |
|  | MCAR-sp | 58.47 | - | - |  | 68.11 | - | - | 126.58 |

PO, presence-only

*Supplementary Figure:*

Figure S1 Location of the analyzed ports. Port abbreviations are based on the United Nations Code for Trade and Transport Locations (UN/LOCODE).


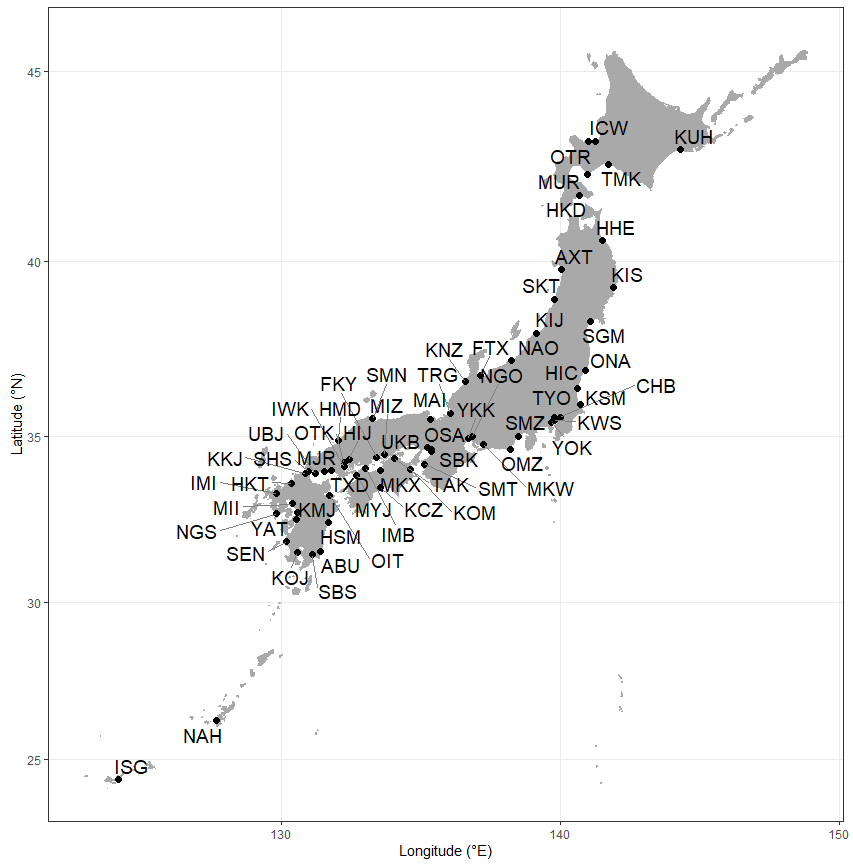


**Appendix S2: Supplement for model selection**

**Strategy**

We applied a stepwise model selection procedure in order to identify the variables that significantly explained the response variable $Y$. We considered the variables of the number of imported containers $X_{NC}$ and the annual minimum temperature $X_{MT}$Initially, a comprehensive model was constructed by incorporating all the covariates deemed potentially influential on the response variable. The full model (CASE1) is described as

$$Y X_{NC}+X_{MT}$$

We tested two reduced models by removing $X_{MT}$, resulting in $Y X_{NC}$ (CASE2) and removing $X_{NC}$, resulting in $Y X_{MT}$ (CASE3). We also checked the results using WAIC. Therefore, the MT variable was removed from the model (Table S6).

Table S5 DIC and WAIC values for model selection

|  |  |  | DIC | | | |  | WAIC | | | |
| --- | --- | --- | --- | --- | --- | --- | --- | --- | --- | --- | --- |
|  |  |  | Survey | PO | Diff. from Full | |  | Survey | PO | Diff. from Full | |
| CASE 1 | Full model |  |  |  |  |  |  |  |  |  |  |
| *S. invicta* | Separate | GLM | 60.44 | 68.29 |  |  |  | 61.19 | 69.97 |  |  |
|  |  | CAR | 60.04 | 68.13 |  |  |  | 60.90 | 69.73 |  |  |
|  | Joint | MCAR-dt | 59.19 | 67.78 |  |  |  | 59.21 | 71.41 |  |  |
|  |  | MCAR-sp | 58.69 |  |  |  |  | 58.83 |  |  |  |
| *S. geminata* | Separate | GLM | 72.28 | 115.71 |  |  |  | 73.83 | 120.80 |  |  |
|  |  | CAR | 71.57 | 107.88 |  |  |  | 73.48 | 126.94 |  |  |
|  | Joint | MCAR-dt | 69.69 | 102.06 |  |  |  | 71.79 | 110.88 |  |  |
|  |  | MCAR-sp | 68.70 |  |  |  |  | 70.96 |  |  |  |
| CASE 2 |  |  |  |  |  | |  |  |  |  | |
| *S. invicta* | Separate | GLM | 60.08 | 66.06 | -0.4 | -2.2 |  | 61.00 | 66.49 | -0.20 | -3.50 |
|  |  | CAR | 59.65 | 65.89 | -0.4 | -2.2 |  | 60.69 | 66.31 | -0.20 | -3.40 |
|  | Joint | MCAR-dt | 58.55 | 65.64 | -0.6 | -2.1 |  | 58.73 | 66.90 | -0.50 | -4.50 |
|  |  | MCAR-sp | 58.55 | NA | -0.1 |  |  | 58.47 |  | -0.40 |  |
| *S. geminata* | Separate | GLM | 70.11 | 113.82 | -2.2 | -1.9 |  | 70.76 | 117.51 | -3.10 | -3.30 |
|  |  | CAR | 69.53 | 107.10 | -2 | -0.8 |  | 70.47 | 126.64 | -3.00 | -0.30 |
|  | Joint | MCAR-dt | 67.62 | 100.22 | -2.1 | -1.8 |  | 68.19 | 104.42 | -3.60 | -6.50 |
|  |  | MCAR-sp | 67.17 |  | -1.5 |  |  | 68.11 |  | -2.80 |  |
| CASE 3 |  |  |  |  |  | |  |  |  |  | |
| *S. invicta* | Separate | GLM | 127.78 | 269.71 | 67.34 | 201.42 |  | 130.39 | 277.59 | 69.20 | 207.62 |
|  |  | CAR | 93.24 | 175.34 | 33.2 | 107.21 |  | 122.76 | 417.29 | 61.86 | 347.56 |
|  | Joint | MCAR-dt | 104.30 | 153.28 | 45.1 | 85.5 |  | 146.98 | 330.43 | 87.77 | 259.02 |
|  |  | MCAR-sp | 81.29 |  | 22.61 |  |  | 94.72 |  | 35.89 |  |
| *S. geminata* | Separate | GLM | 125.12 | 277.76 | 52.84 | 162.05 |  | 127.27 | 286.77 | 53.44 | 165.97 |
|  |  | CAR | 97.38 | 173.73 | 25.82 | 65.85 |  | 131.58 | 374.84 | 58.10 | 247.90 |
|  | Joint | MCAR-dt | 99.22 | 150.86 | 29.53 | 48.8 |  | 125.16 | 264.49 | 53.37 | 153.61 |
|  |  | MCAR-sp | 79.52 |  | 10.82 |  |  | 85.17 |  | 14.21 |  |

Abbreviations: DIC, deviance information criterion; WAIC, Watanabe-Akaike information criterion; CAR, conditional autoregressive models; MCAR, multivariate CAR; PO, presence-only

**Appendix S3: Supplement for model implementation**

**Jacobian of a variable transformation**

In the INLA, the hyperparameters are represented as an internal scale to simplify the numerical optimization. It is recommended that the internal scale parameter is not bounded because this simplifies the computations. The *“*rgeneric*”* model implemented in INLA requires defining the function which returns the log prior density for internal scale $\theta$. Therefore, a Jacobian must be added to the log-prior density.

The precision matrix $\mathbf{Q}$ of the bivariate Leroux model is specified as follows:

$\mathbf{Q}=\boldsymbol{\Lambda}\otimes\left[ \left( 1-\lambda\right)I+\lambda\{\mathrm{diag}\left( \mathbf{W}1 \right)-\mathbf{W}\} \right]$.

The hyperparameters were transformed to the internal scale $\theta$ as follows:

$$\theta=\log\left( \tau\right)$$

$$\theta=\mathrm{logit}\left( \frac{\rho+1}{2} \right)$$

$$\theta=\mathrm{logit}\left( \lambda\right)\text{.}$$

Each Jacobian is expressed as follows:

***Precision*** $\tau$

Assuming that the prior precision $\tau$ distribution follows a gamma distribution with shape parameter $a$ and rate parameter $b$, the probability density function of $\tau$ is given by:

$$f\left( \tau;a,b \right)=\text{Gamma}\left( a,b \right)=\frac{b^{a}}{\Gamma\left( a \right)}\tau^{a-1}e^{-b\tau},\text{for }\tau>0.$$

Considering the variable transformation $\theta=log\left( \tau\right)$, the log prior needs to be provided on the internal scale ($\theta$) instead of the parameter scale ($\tau$). As $\tau=e^{\theta}$, the Jacobian is given by:

$$\frac{d\tau}{d\theta}=\frac{d}{d\theta}\left( e^{\theta} \right)=e^{\theta}.$$

The density function on the internal scale is

$$f\left( \theta;a,b \right)=f\left( \tau;a,b \right)\frac{d\tau}{d\theta}=\text{Gamma}\left( a,b \right)\cdot e^{\theta}.$$

Therefore, the log prior density on the internal scale is the log density on the parameter scale plus $\log\left( e^{\theta} \right)=\theta$.

***Correlation*** $\rho$

The correlation parameter was transformed as $\theta=\mathrm{logit}\left( \frac{\rho+1}{2} \right)$. As $\rho=\frac{e^{\theta}-1}{1+e^{\theta}}$, Jacobian is given by:

$$\frac{d\rho}{d\theta}=\frac{d}{d\theta}\left( \frac{e^{\theta}-1}{1+e^{\theta}} \right)$$

$$=\frac{e^{\theta}\left( 1+e^{\theta} \right)-\left( e^{\theta}-1 \right)e^{\theta}}{\left( 1+e^{\theta} \right)^{2}}$$

$$=\frac{2e^{\theta}}{\left( 1+e^{\theta} \right)^{2}}.$$

The following is then added to the log density of the parameter scale:

$$\log\left( \frac{2e^{\theta}}{\left( 1+e^{\theta} \right)^{2}} \right)=\log\left( 2 \right)+\log\left( e^{\theta} \right)-2\log\left( 1+e^{\theta} \right)\text{.}$$

***Spatial dependence*** $\lambda$

The spatial dependence parameter $\lambda$ was transformed as $\theta=\mathrm{logit}\left( \lambda\right)=\log\left( \frac{\lambda}{1-\lambda} \right)$. As $\lambda=\frac{1}{1+e^{-\theta}}$, Jacobian is given by:

$$\frac{d\lambda}{d\theta}=\frac{d}{d\theta}\left( \frac{1}{1+e^{-\theta}} \right)$$

$$=\frac{e^{-\theta}}{\left( 1+e^{-\theta} \right)^{2}}$$

$$=\frac{1}{1+e^{-\theta}}\frac{e^{-\theta}}{1+e^{-\theta}}$$

$$=\lambda\left( 1-\lambda\right).$$

The following is then added to the log density of the parameter scale:

$$\log\left( \lambda\left( 1-\lambda\right) \right)=\log\left( \lambda\right)+\log\left( 1-\lambda\right)\text{.}$$
